# Cerebral Microvascular Endothelial Cell Apoptosis after Ischemia: Role of Enolase-Phosphatase 1 Activation and Aci-Reductone Dioxygenase 1 Translocation

**DOI:** 10.3389/fnmol.2016.00079

**Published:** 2016-08-31

**Authors:** Yuan Zhang, Ting Wang, Ke Yang, Ji Xu, Lijie Ren, Weiping Li, Wenlan Liu

**Affiliations:** ^1^The Central Laboratory, Shenzhen Second People’s Hospital, Graduate School of Guangzhou Medical UniversityShenzhen, China; ^2^Shenzhen Key Laboratory of Neurosurgery, Shenzhen Second People’s Hospital, Graduate School of Guangzhou Medical UniversityShenzhen, China; ^3^Department of Pathophysiology, Baotou Medical CollegeBaotou, China; ^4^Department of Neurology, Shenzhen Second People’s Hospital, The First Affiliated Hospital of Shenzhen UniversityShenzhen, China; ^5^Department of Neurosurgery, Shenzhen Second People’s Hospital, The First Affiliated Hospital of Shenzhen UniversityShenzhen, China

**Keywords:** ENOPH1, oxygen-glucose deprivation, cerebral ischemia, blood brain barrier, apoptosis

## Abstract

Enolase-phosphatase 1 (ENOPH1), a newly discovered enzyme of the methionine salvage pathway, is emerging as an important molecule regulating stress responses. In this study, we investigated the role of ENOPH1 in blood brain barrier (BBB) injury under ischemic conditions. Focal cerebral ischemia induced ENOPH1 mRNA and protein expression in ischemic hemispheric microvessels in rats. Exposure of cultured brain microvascular endothelial cells (bEND3 cells) to oxygen-glucose deprivation (OGD) also induced ENOPH1 upregulation, which was accompanied by increased cell death and apoptosis reflected by increased 3-(4, 5-Dimethylthiazol-2-yl)-2, 5- diphenyltetrazolium bromide formation, lactate dehydrogenase release and TUNEL staining. Knockdown of ENOPH1 expression with siRNA or overexpressing ENOPH1 with CRISPR-activated plasmids attenuated or potentiated OGD-induced endothelial cell death, respectively. Moreover, ENOPH1 knockdown or overexpression resulted in a significant reduction or augmentation of reactive oxygen species (ROS) generation, apoptosis-associated proteins (caspase-3, PARP, Bcl-2 and Bax) and Endoplasmic reticulum (ER) stress proteins (Ire-1, Calnexin, GRP78 and PERK) in OGD-treated endothelial cells. OGD upregulated the expression of ENOPH1’s downstream protein aci-reductone dioxygenase 1 (ADI1) and enhanced its interaction with ENOPH1. Interestingly, knockdown of ENOPH1 had no effect on OGD-induced ADI1 upregulation, while it potentiated OGD-induced ADI1 translocation from the nucleus to the cytoplasm. Lastly, knockdown of ENOPH1 significantly reduced OGD-induced endothelial monolayer permeability increase. In conclusion, our data demonstrate that ENOPH1 activation may contribute to OGD-induced endothelial cell death and BBB disruption through promoting ROS generation and the activation of apoptosis associated proteins, thus representing a new therapeutic target for ischemic stroke.

## Introduction

Ischemic stroke is a leading cause of disability and mortality in humans. Cerebral ischemia initiates a cascade of cytotoxic molecules responsible for the death of neural cells as well as the damage of the blood brain barrier (BBB) at the injury site. The polyamines, such as putrescine, spermindine and spermine, are elevated in the ischemic parenchyma and contribute to ischemic brain damage via enhancing N-methy-D-aspartate receptor-mediated excitotoxicity, generating toxic aldehydes and reactive oxygen species (ROS), and disrupting oxidative metabolism and mitochondrial function (Takano et al., 2005; Kim et al., 2009). The administration of polyamine antagonists prevents the development of ischemic brain damage (Takano et al., 2005; Li et al., 2007).

Enolase-phosphatase 1 (ENOPH1) is a newly identified enzyme of the methionine salvage pathway, a ubiquitous pathway for the reconstitution of methionine, which is required for the synthesis of polyamine (Sauter et al., 2013). A recent study shows that ENOPH1 is widely expressed in the brain and stress exposure increases ENOPH1 protein levels in brain tissue of C57BL/6J mice (Barth et al., 2014). Since ENOPH1 participates in the synthesis of polyamine indirectly via S-adenosyl methionine (SAM; Takano et al., 2005; Li et al., 2007; Kim et al., 2009; Duan et al., 2011), it is logical to speculate a role of ENOPH1 in ischemic brain injury.

**Figure 1:**
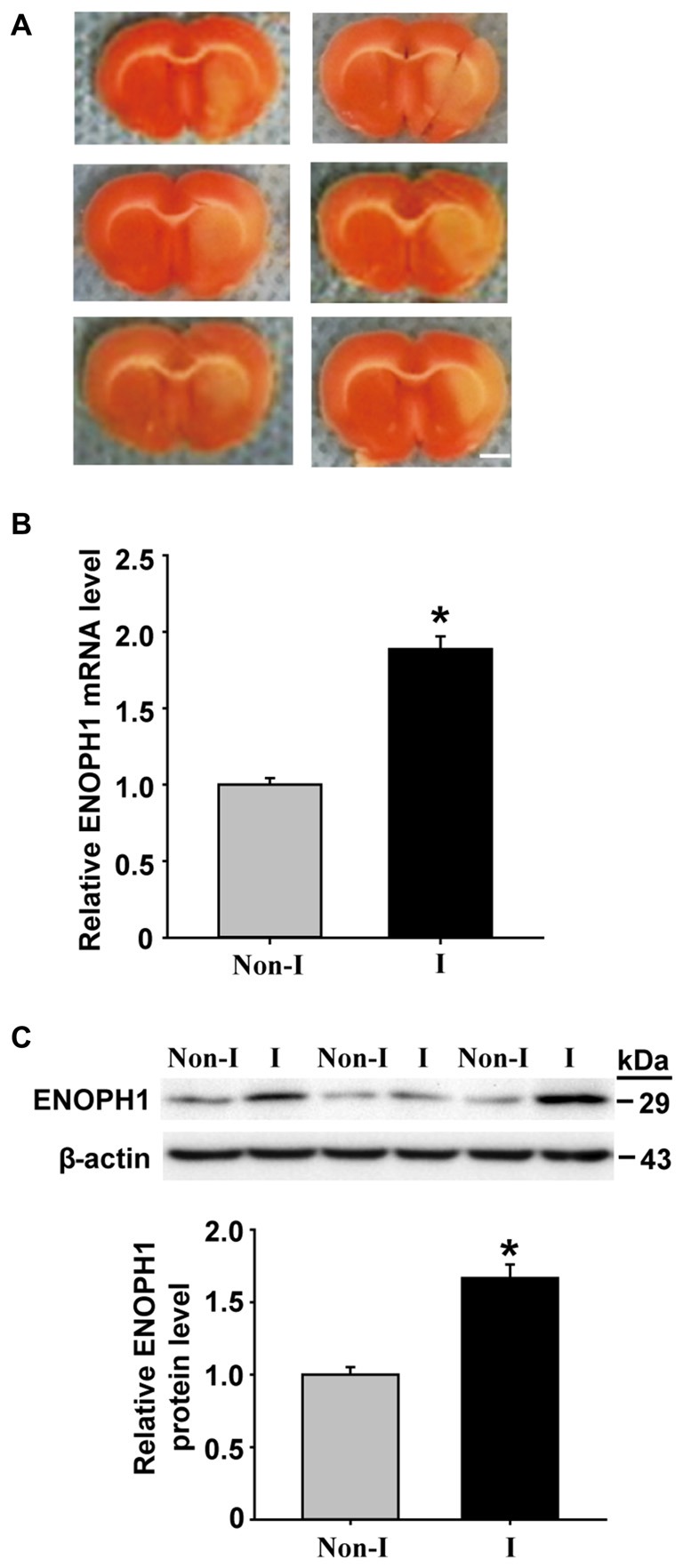
**Middle cerebral artery occlusion (MCAO) induces Enolase-phosphatase 1 (ENOPH1) upregulation in ischemic cerebral microvessels.** Rats were subjected to 3 h MCAO before isolating hemispheric cerebral microvessels. The mRNA and protein levels of ENOPH1 in cerebral microvessels from nonischemic (Non-I) and ischemic (I) hemispheric tissue were analyzed by real-time RT-PCR and western blot. **(A)** Representative photographs of triphenyltetrazolium chloride (TTC) stained 1 mm-thick brain sections showing tissue infarction (pale white region) in the ischemic hemispheres (right). **(B)** Real-time RT-PCR analysis showed that ENOPH1 mRNA expression was significantly increased in ischemic hemispheric microvessels. *P < 0.05 vs. Non-I; *n* = 6. **(C)** Western blot analysis revealed increased levels of ENOPH1 protein in ischemic hemispheric microvessels. Upper panel: representative immunoblots of ENOPH1 and the loading control β-actin; bottom panel: quantitative data of protein band intensity after normalization to β-actin. *P < 0.05 vs. Non-I; *n* = 6.

BBB disruption is a common event in ischemic stroke, which leads to vasogenic brain edema and hemorrhagic transformation (Sandoval and Witt, 2008; Hawkins et al., 2014). Brain capillary endothelial cells form the backbone of the BBB structure, their death or apoptosis can result in catastrophic failure of BBB’s integrity (ElAli et al., 2011; Wang et al., 2011). We have obtained preliminary data showing that cerebral ischemia induces ENOPH1 mRNA expression in ischemic cerebral microvessels in a rat model of middle cerebral artery occlusion (MCAO; Figure 1). However, the exact role of ENOPH1 in BBB damage remains to be determined.

In this study, we applied *in vitro* cultured brain microvascular endothelial monolayer and oxygen-glucose deprivation (OGD) to mimic the BBB and ischemia, respectively, and explored the role of ENOPH1 in ischemic BBB damage. Our data showed that OGD induced a significant increase in the protein levels of ENOPH1 and its downstream molecule aci-reductone dioxygenase 1 (ADI1) in cultured brain endothelial cells, which led to increased ROS generation, endothelial cell apoptosis and increased permeability of endothelial monolayer.

## Materials and Methods

### Rat Model of MCAO

The Laboratory Animal Care and Use Committee of Shenzhen University approved all experimental protocols. Male Sprague Dawley rats (Southern Medical University, Guangzhou, Guangdong, China) weighing 290–320 g were anesthetized with isoflurane (4% for induction, 1.75% for maintenance) in N_2_O:O_2_ (70%:30%) during surgical procedures and the body temperature was maintained at 37.5 ± 0.5°C using a heating pad. The rats were subjected to 3 h MCAO using the intraluminal filament technique as previously described (Liu et al., 2009). Briefly, a 4–0 silicone-coated monofilament nylon suture was inserted into the internal carotid artery and advanced along the internal carotid artery to approximately 17–18 mm from the bifurcation, thereby blocking the ostium of the MCA. At the end of 3 h MCAO, rats were sacrificed and the brains were removed for microvessel isolation as described below. For a total number of 12 rats included in this study, successful MCAO was confirmed by 2,3,5-triphenyltetrazolium chloride (TTC) staining of the 1 mm-thick brain coronal section 6 mm away from the tip of the front lobe as we described previously (Liu et al., 2008).

### Isolation of Cerebral Microvessels

Isolation of cerebral microvessels was carried out as we described previously (Liu et al., 2009). Briefly, the hemispheric brain tissue was dissected and homogenized in ice cold Phosphate Buffered Saline (PBS). The homogenate was filtered through a 41 μm nylon mesh (Spectrum), and the nylon mesh was washed three times with PBS. Microvessels retained on the mesh were then washed off and pelleted by centrifugation at 4000 g for 10 min at 4°C. The pellets were resuspended in 15% dextran T-500 and then added onto 20% dextran T-500, followed by centrifugation at 25,000 g for 10 min at 4°C. The pellets were collected at the cerebral microvessels and used for measuring ENOPH1 mRNA and protein expression by real time RT-PCR and western blot, respectively.

### Cell Cultures

Mouse brain microvascular endothelial cells (bEND3 cells; American Type Culture Collection, VA, USA) were grown as a monolayer in Dulbecco’s modified Eagle’s medium (DMEM) with 10% fetal bovine serum (FBS), 100 U/ml penicillin and 100 μg/ml streptomycin at 37°C in a humidified incubator gassed with 5% CO_2_ and 95% room air. The cells were grown to confluence on type I collagen-coated 60 mm dishes before exposure to OGD. For the assays, cell cultures were initiated at a density of 5 × 10^5^ cells/ml to have cells in the exponential growth phase. The number of cells was determined with a hemocytometer (Adam MC, Digital bio, Korea).

### OGD Treatment

To mimic ischemic condition *in vitro*, bEND3 cells were exposed to OGD as described previously (Liu et al., 2012). In brief, confluent bEND3 cells were subjected to OGD by replacing the normal growth medium with glucose free medium (DMEM without glucose) pre-equilibrated with 95% N_2_ and 5% CO_2_. The cells were then incubated in a humidified airtight chamber (Biospherix Ltd., Lacona, NY, USA) for 1, 3 or 6 h. Control cultures were incubated with normal DMEM medium without FBS at 37°C in 5% CO_2_/95% air. The cells were collected for further analyses immediately after OGD treatment.

### Lactate Dehydrogenase Release Assay

After OGD treatment, cell viability was measured using a CytoTox 96^®^Non-Radioactive Cytotoxicity Assay Kit (Promega) according to manufacturer’s instruction. Briefly, 50 μl of each sample medium (e.g., pure culture medium for measuring background LDH release, culture media collected from control or OGD-treated cells for measuring experimental LDH release and lysis buffer-treated cells for measuring maximum LDH release) was collected to assay LDH release. The samples were incubated with the reduced form of nicotinamide-adenine dinucleotide and pyruvate for 30 min at room temperature and the reaction was terminated by adding Stop Solution. LDH release was assessed by measuring the absorbance of supernatants at 490 nm. The cell death rate was calculated as follows: cell death rate = (experimental LDH release − background LDH release)/(maximum LDH release-background LDH release) × 100%. The results were presented as fold increase of the control cells.

### Knockdown of ENOPH1 with siRNA

bEND3 cells at 60–70% confluence on 6- and 24-well plates were transfected with 100 nM ENOPH1 siRNA (si-ENOPH1, sc-144654, Santa Cruz, CA, USA) or scrambled control siRNA (si-control, sc-37007, Santa Cruz, CA, USA) using siRNA transfection reagent (sc-29528) according to the manufacturer’s instruction. Forty-eight hours after transfection, cells were subjected to OGD treatment. Specific silencing was confirmed by western blot.

### ENOPH1 CRISPR Activation Plasmid Transfection

bEND3 cells grown to 60–80% confluence were transfected with the ENOPH1 overexpression plasmid (ENOPH1 CRISPR activation plasmid, Santa Cruz, CA, USA) or control plasmid (control CRISPR activation plasmid, Santa Cruz, CA, USA) using UltraCruz^®^ Transfection Reagent (Santa Cruz Biotech) according to the manufacturer’s instruction. In brief, for each transfection, a 300 μl mixture of 1 μg of plasmid DNA with 10 μl of UltraCruz^®^ transfection reagent in plasmid transfection medium was added to each well. Forty-eight hours after transfection, cells were subjected to OGD treatment and subjected to various assays.

### *In vitro* BBB Model of Endothelial Monolayer

An endothelial monolayer grown on a cell culture insert is widely used for *in vitro* BBB models. Nunc cell culture inserts (for 24-well plates) with 0.02 μm Anapore membranes (Nunc Inc., Naperville, IL, USA) were coated by incubation with 70 μg/ml type I collagen (Sigma, St. Louis, CA, USA) in 20 mM acetic acid for 1 h at 25°C. The inserts were then washed with serum free medium to remove excess protein before adding complete medium to equilibrate the membrane for 3 h at 37°C in a cell culture incubator. Then, the endothelial cells were trypsinized from the tissue culture flasks, washed three times with complete medium, and seeded on the inserts at 20,000 cells/cm^2^. The cells seeded on inserts were allowed to grow for 3–4 days at 37°C in 5% CO_2_/95% air to achieve full confluence, which was confirmed under a phase contrast microscopy.

### Endothelial Cell Monolayer Permeability Assay

Endothelial monolayer permeability after OGD was measured as we described previously (Liu et al., 2012). Briefly, bEND3 cells were placed on the upper side of the insert and allowed to grow to confluence. Then, 3.5 μM fluorescein isothiocyanate (FITC)-dextran was added to the endothelial monolayer (luminal compartment) before expose to OGD for 6 h. After OGD treatment, the contents of FITC-dextran in both luminal and abluminal compartments were determined and endothelial monolayer permeability was assessed by calculating the apparent permeability coefficient (Papp) as previously described (Grabovac and Bernkop-Schnürch, 2006): Papp [cm/s] = dQ/(dt*A*Co), where dQ was the amount of FITC-dextran getting into the abluminal compartment, dt was duration of OGD treatment, dQ/dt was the rate of transfer (ng/s), A was surface area (cm^2^), and Co was the initial concentration in the luminal chamber (ng/cm^3^). To test whether ENOPH1 were implicated in OGD-induced endothelial barrier disruption, cells were pretreated with ENOPH1 siRNA for 48 h before OGD treatment.

#### Detection of Intracellular Reactive Oxygen Species (ROS) Generation

Intracellular ROS generation was assessed by quantifying fluorescence intensity under a fluorescent microscope or by flow cytometry analysis after staining with 2,7-dichlorodihydrofluorescein diacetate (DCF-DA). The bEND3 cells were pre-incubated in a 24- or 6-well culture plate for 24 h. Subsequently, the cells were transfected with control or ENOPH1 siRNA for 48 h at 37°C. After transfection, the bEND3 cells were treated with OGD for 6 h before incubating with 10 μM DCF-DA (Sigma, St. Louis, MO, USA) for 30 min at 37°C. The cells were then photographed under a fluorescent microscope (Leica, Germany) or resuspended and incubated for another 10 min with propidium iodide (10 μg/ml) before analyzing ROS with a Fluorescence-activated cell sorting (FACS) calibur flow cytometer (Becton Dickinson, excitation: 488 nm). ROS fluorescence was quantified and expressed as fold increase of the control cells.

#### Western Blot Assay

After indicated treatment, the cells grown on 6 or 10 cm culture dish were harvested and lysed in a cold lysis buffer [50 mM Tris (pH 7.4), 150 mM NaCl, 2 mM Ethylene diamine tetraacetic acid (EDTA), 10% glycerol, 1% TritonX-100, 1 mM phenylmethylsulfonyl fluoride (PMSF) and 2% protease inhibitor mixture (Sigma, St. Louis, MO, USA)]. The cell lysates were centrifuged for 15 min at 12,000 g at 4°C and protein concentrations were determined using Pierce^TM^ BCA Protein Assay Kit (Thermo Fisher Scientific Inc, Waltham, MA, USA). The cell lysates (30 μg protein/lane) were subjected to electrophoresis on 10–12% sodium dodecyl sulfate (SDS)-polyacrylamide gels and transferred to polyvinylidene difluoride membrane. The membranes were blocked in Trisbuffered saline with 0.05% Tween-20 (TBS-T) containing 5% nonfat milk and blotted overnight with primary antibodies (dilution: 1:1000). The membranes were washed and incubated for 1 h with horseradish peroxidase (HRP)-conjugated secondary antibodies (dilution: 1:1000, Jackson ImmunoResearch, West Grove, PA, USA). The membranes were washed and developed using a chemiluminescence kit (Fisher Scientific). The primary antibodies were cleaved-caspase-3, caspase-3, cleaved-PARP, Bcl-2, Bax, PARP, ADI1, PERK, Calnexin, Ire-1α, ENOPH1 and β-actin. ENOPH1 antibody was a product of Proteintech (Cat. Log: 11763-1-AP), and the rest primary antibodies were purchased from Santa Cruz Biotech. Relative protein levels were normalized to β-actin. For subcellular fraction samples, we noticed that the actin levels were comparable for each sample among different fractions, so we only used one normalizing loading control (CF actin) for all three subcellular fractions.

#### Coimmunoprecipitation of ENOPH1 with ADI1

Coimmunoprecipitation was performed as described previously (Wen et al., 2010). Briefly, bEND3 cells were subjected to OGD treatment for 6 h and then lysed on ice in 1 ml RIPA buffer. After pre-clearing with normal IgG, cell lysates (0.5 mg of protein) were incubated overnight at 4°C with 2 μg of anti-ENOPH1 (Proteintech, Chicago, IL, USA), followed by precipitation with 20 μl of protein A/G Plus-Agarose (Pierce Biotechnology, Rockford, IL, USA) for 1 h at 4°C. The precipitated complexes were separated on SDS-PAGE gels and immunoblotted with anti-ADI1 (Santa Cruz Biotech., Dallas, TX, USA) to detect the presence of this protein in the complex, as described above.

#### Immunocytochemistry

bEND3 cells grown on type I collagen-coated coverslips were exposed to OGD for 6 h before fixation with 4% paraformaldehyde and permeabilization with 0.1% Triton X-100. After blocking non-specific binding with a blocking solution (3% BSA, 0.1% Tween 20 and 5% goat serum in PBS), the cells were incubated overnight at 4°C with anti-ENOPH1 (Proteintech, 1:200) primary antibodies, then followed by FITC anti-mouse secondary antibodies (Invitrogen, 1:200) for 1 h at room temperature. Coverslips were mounted on glass slides using anti-fade sealed solution (Beyotime Biotechnology, Jiangsu, China) and immunostaining was visualized under a DMI6000B fluorescence microscope (Leica, Germany). The average fluorescence intensity of single nuclei (Iav) and the area (A) that they occupied in the image were determined and the integrated optical density (IOD), which is proportional to the amount of incorporated ENOPH1 antibody, was calculated by the formula: IOD = Iav *A.

#### Real-Time RT-PCR

Total RNA was isolated from endothelial cells using Trizol reagent (Invitrogen, Carlsbad, CA, USA). RNA samples (2 μg) were reverse-transcribed to generate first-strand cDNA. After reverse transcription using TaqMan^®^ Reverse Transcription Kits (Applied Biosystems), 0.5 μl reverse-transcribed products were amplified with the Vii7 real-time PCR System (Applied Biosystems) in a 10 μl final reaction volume using SYBR^®^ Green PCR Master Mix (Applied Biosystems) under the following conditions: 30 s at 95°C, followed by a total of 40 cycles of two temperature cycles (15 s at 95°C and 1 min at 60°C). Primer sequences were as follows: rat ENOPH1 forward: 5′-ACCACAACCCCGATTGCTTT-3′ and reverse: 5′-TTCTTCAGCCTGCTTCCTCA-3′; mouse ENOPH1 forward: 5′-ACCACAACCCCGATTGCTTT-3′ and reverse: 5′-TTCCTCGGCCTGTTTCCTCA-3′; mouse ADI1 forward: 5′-CCGAATGGAAAG TTGCTC-3′ and reverse: 5′-TAAGTCTTGACAGTTAGGGA-3′; GAPDH served as endogenous control, and the primers were forward: 5′-CAATGTGTCCGTCGTGGAT CT-3′; reverse:5′-GTCCTCAGTGTAGCCCAAGATG-3′. The Ct value was calculated by the comparative ΔΔC_t_ method using the SDS Enterprise Database software (Applied Biosystems).

#### TUNEL Assay

Apoptosis was analyzed by TUNEL assay using Click-iT^®^ Plus TUNEL Assay (Life Technologies, Inc., Carlsbad, CA, USA) according to manufacturer’s instruction. In brief, at the end of the indicated treatments, bEND3 cells grown on coverslips were incubated with TdT reaction mixture for 2 h at 37°C, followed by 30 min incubation with the Alexa Fluor^®^ 594 dye (red fluorescence). Then, the cells were counterstained with DAPI (Sigma-Aldrich) for 20 min and observed under a fluorescence microscope (magnification, ×200; Leica, Germany). The TUNEL-positive nuclei of six non-overlapping fields per coverslip were counted by a researcher blinded to treatment, and these counts were converted to percentages by comparing the TUNEL-positive counts to the total number of cell nuclei as determined by DAPI counterstaining, that is TUNEL-positive ratio = (number of red nuclei/number of blue nuclei) × 100%.

#### Statistical Analysis

All data were expressed as means ± SEM. Differences between groups were evaluated by either an unpaired Student’s *t*-test or one-way analysis of variance (ANOVA) followed by Tukey’s *post hoc* test. A value of *P* < 0.05 was considered statistically significant.

## Results

### ENOPH1 is Upregulated in Ischemic Cerebral Microvessels

ENOPH1 is found to be widely expressed in the brain and is implicated in stress response (Barth et al., 2014). To determine whether ENOPH1 plays a role in ischemic BBB injury, we first examined the change of ENOPH1 expression in the BBB, i.e., the cerebral microvessels or capillaries, isolated from the rats that were subjected to 3 h MCAO without reperfusion. TTC staining of the 1 mm-thick brain sections collected from the six rats included in this study showed visible tissue infarction (no TTC staining, white color) in the MCA supplied area of the right hemisphere after 3 h MCAO (Figure 1A). ENOPH1 mRNA expression was analyzed in isolated hemispheric microvessels by real time RT-PCR and found that 3 h MCAO induced a significant increase (~1-fold) of ENOPH1 mRNA expression in ischemic hemispheric microvessels compared to nonischemic microvessels tissue (Figure 1B). Consistent with its mRNA change, ENOPH1 protein levels were also significantly increased in ischemic cerebral microvessels (Figure 1C). These results demonstrate that ENOPH1 is upregulated in the ischemic brain microvessels. To further demonstrate the role of ENOPH1 in ischemic BBB injury and the underlying mechanisms involved, we chose the widely used *in vitro* model of BBB (i.e., brain microvascular endothelial cell monolayer) and the *in vitro* model of ischemia (i.e., OGD) for the rest of this study.

### OGD Induces ENOPH1 Expression in Brain Endothelial Cells

**Figure 2:**
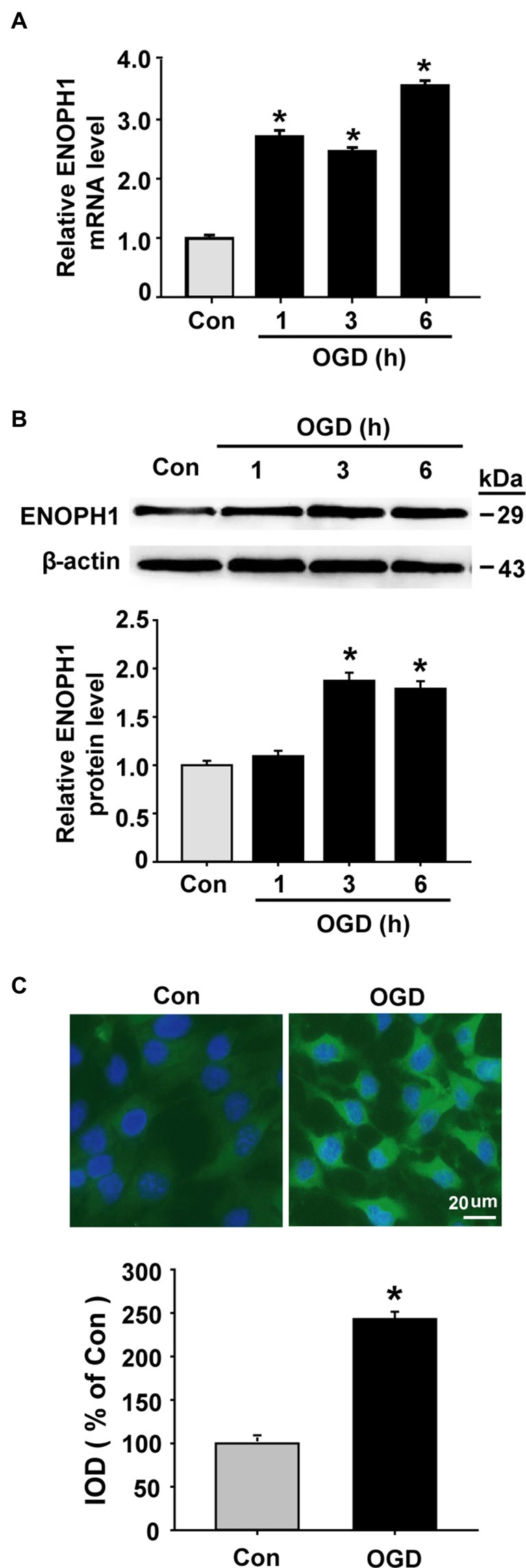
**Oxygen-glucose deprivation (OGD) induces upregulation of ENOPH1 in brain microvascular endothelial cells (bEND3 cells).** bEND3 cells were subjected to OGD treatment or normoxia (Control, Con) for 1, 3, or 6 h before analyzing ENOPH1 mRNA and protein expression using real-time RT-PCR, western blot and immunostaining. **(A)** Real time RT-PCR analysis showed that ENOPH1 mRNA expression was significantly increased in bEND3 cells at 1 h after OGD treatment and was further increased when OGD was prolonged to 6 h. *P < 0.05 vs. Con; *n* = 4. **(B)** Western blot analysis showed that ENOPH1 protein levels were increased in 3 h OGD and 6 h OGD treated cells, but not in 1 h OGD treated cells. Upper panel: representative immunoblots of ENOPH1 and the loading control β-actin; bottom panel: quantitative data of protein band intensity after normalization to β-actin., *P < 0.05 vs. Con; *n* = 4. **(C)** bEND3 cells were exposed to OGD for 6 h before immunostaining with ENOPH1 (green), and nuclei were counterstained with DAPI (blue). Upper panel: representative immunocytochemical micrographs showed that ENOPH1 was located in the cytosol and OGD significantly enhanced ENOPH1’s fluorescence intensity (bar = 20 μm); bottom panel: the intensities of ENOPH1 immunofluorescence were quantitated and expressed as IOD parameter. *P < 0.05 vs. Con; *n* = 4. IOD, integrated optical density.

As mentioned earlier, ENOPH1 is a newly identified protein and little is known about its biological function. So far, there are no specific pharmacological inhibitors or genetics-manipulated animals available, therefore we chose the widely used *in vitro* model of BBB (i.e., cultured endothelial monolayer) and the *in vitro* model of ischemia (i.e., OGD) to investigate whether ENOPH1 plays a role in ischemic BBB injury. Brain endothelial cells bEND3 were exposed to OGD for 1, 3, or 6 h before analyzing ENOPH1 mRNA and protein levels. Real time RT-PCR analysis showed that ENOPH1 mRNA expression was increased in bEND3 cells after exposing to OGD for 1 h and was further increased at 6 h OGD, while no significant difference was seen between 1 h and 3 h OGD (Figure 2A). Western blot analysis showed that ENOPH1 protein levels were significantly increased in bEND3 cells after exposing to OGD for 3 h, but not for 1 h (Figure 2B). Different from the mRNA result, 6 h OGD did not further increase ENOPH1 protein levels in bEND3 cells compared to 3 h OGD (Figure 2B). To further verify the immunoblot findings and to reveal ENOPH1’s intracellular localization, we performed immunocytochemical staining after exposing bEND3 cells to OGD for 6 h. As shown in Figure 2C, immunostaining revealed that ENOPH1 protein was located in the cytosol of bEND3 cells and OGD treatment significantly increased ENOPH1’s fluorescence intensity (~1.5-fold increase) compared to control (normoxic) cells. These data demonstrate that OGD significantly induces ENOPH1 upregulation in brain endothelial cells.

### Knockdown of ENOPH1 Inhibits OGD-Induced Endothelial Cell Apoptosis

**Figure 3:**
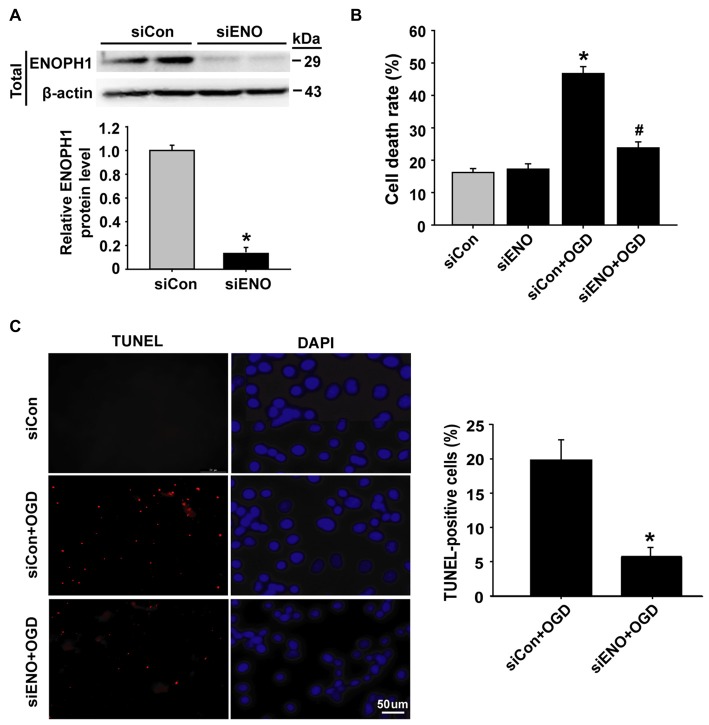
**Knockdown of ENOPH1 attenuates OGD-induced apoptosis in bEND3 cells. (A)** ENOPH1 siRNA effectively knocked down ENOPH1 protein expression in bEND3 cells. Western blot analysis showed that incubation bEND3 cells with ENOPH1 siRNA (siENO) for 48 h significantly (~90%) reduced ENOPH1 protein levels. Upper panel: representative immunoblots of ENOPH1 and the loading control β-actin; bottom panel: quantitative data of protein band intensity after normalization to β-actin. *P < 0.05 vs. Control siRNA (siCon); *n* = 4. **(B)** Knockdown of ENOPH1 significantly reduced 6 h OGD-induced cell death assessed by LDH release. *P < 0.05 vs. siCon; ^#^*P* < 0.05 vs. siCon + OGD; *n* = 4.** (C)** TUNEL assay showed that 6 h OGD significantly increased the number of TUNEL-positive apoptotic endothelial nuclei (red fluorescence) and knockdown of ENOPH1 significantly decreased this increase. Left panel: representative micrographs of double staining of TUNEL and DAPI (blue, counter staining), bar = 50 μm; right panel: quantitative data of TUNEL-positive cells. *P < 0.05 vs. siCon + OGD; Experiments were repeated four times (*n* = 4).

To determine whether ENOPH1 contributes to ischemia-induced BBB injury, we applied siRNA approach to knock down ENOPH1 expression in bEND3 cells and assessed its effect on 6 h OGD-induced endothelial apoptosis. The efficacy of ENOPH1 siRNA in knocking down ENOPH1 expression was shown in Figure 3A, in which a ~90% reduction in ENOPH1 protein level was seen on immunoblots at 48 h after ENOPH1 siRNA transfection. The effect of ENOPH1 siRNA on OGD-induced endothelial cell death was assessed by measuring LDH release (indicating late apoptosis and necrosis) and TUNEL staining (indicating apoptosis). As shown in Figure 3B, 6 h OGD significantly induced endothelial cell death, with a death rate of 46.74% ± 2.15 vs. 16.19% ± 1.24 of the control cells. Knockdown of ENOPH1 with siRNA had no effect on LDH release from control bEND3 cells, while it significantly reduced OGD-induced cell death. TUNEL staining showed a similar protective effect of ENOPH1 siRNA on OGD-induced apoptosis, with 19.98% ± 2.03 TUNEL-positive cells for OGD alone vs. 7.14% ± 0.95 for OGD plus ENOPH1 siRNA (Figure 3C).

**Figure 4:**
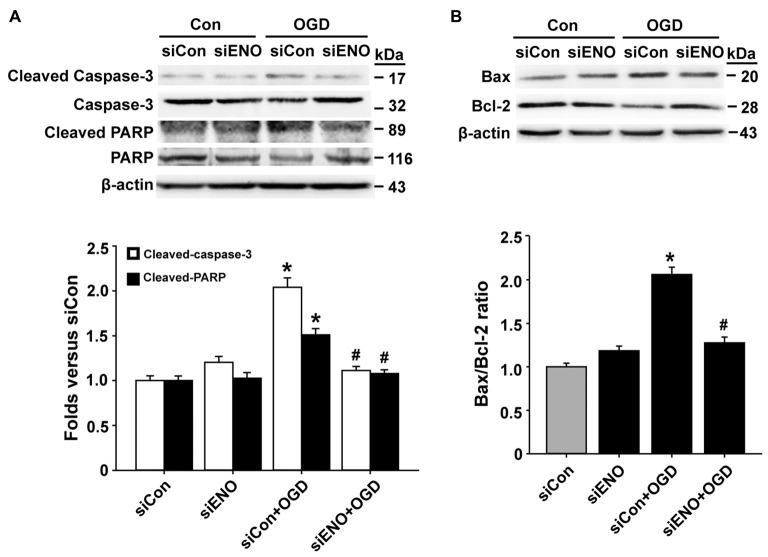
**Knockdown of ENOPH1 attenuates OGD-induced apoptosis-related protein expression in bEND3 cells.** bEND3 cells transfected with siENOPH1 (siENO) or control siRNA (siCon) before exposing to OGD for 6 h. Apoptosis-related proteins were analyzed by western blot. **(A)** Upper panel: representative immunoblots showing the changes of cleaved and full length caspase-3 and PARP protein bands in bEND3 cells. β-actin was used as a loading control; bottom panel: quantitative data showed that OGD significantly increased the cleavage of caspase-3 and PARP and knockdown of ENOPH1 inhibited this change. *P < 0.05 vs. siCon; ^#^*P* < 0.05 vs. siCon + OGD; *n* = 4. **(B)** Upper panel: representative immunoblots of Bax and Bcl-2 proteins; bottom panel: quantitative data showed that OGD significantly increased the ratio of Bax/Bcl-2 and knockdown of ENOPH1 inhibited this increase. *P < 0.05 vs. siCon; ^#^*P* < 0.05 vs. siCon + OGD; *n* = 4.

To further verify a role of ENOPH1 in OGD-induced bEND3 cell apoptosis, we assessed the effect of ENOPH1 siRNA on several key apoptosis-associated signal proteins including caspase-3, PARP and Bax/Bcl-2. As shown in Figures 4A,B, 6 h OGD induced increased levels of cleaved caspase-3 (caspase-3 activation), cleaved PARP-1 and higher ratio of Bax/Bcl-2 in bEND3 cells, and transfection with ENOPH1 siRNA, but not control siRNA, abolished these changes. As expected, ENOPH1 siRNA alone did not affect these apoptosis-associated signal proteins under control conditions (Figures 4A,B). Taken together, these results clearly indicate that ENOPH1 is involved in OGD-induced apoptosis in brain endothelial cells.

### Knockdown of ENOPH1 Abolishes OGD-Induced Oxidative Stress in bEND3 Cells

**Figure 5:**
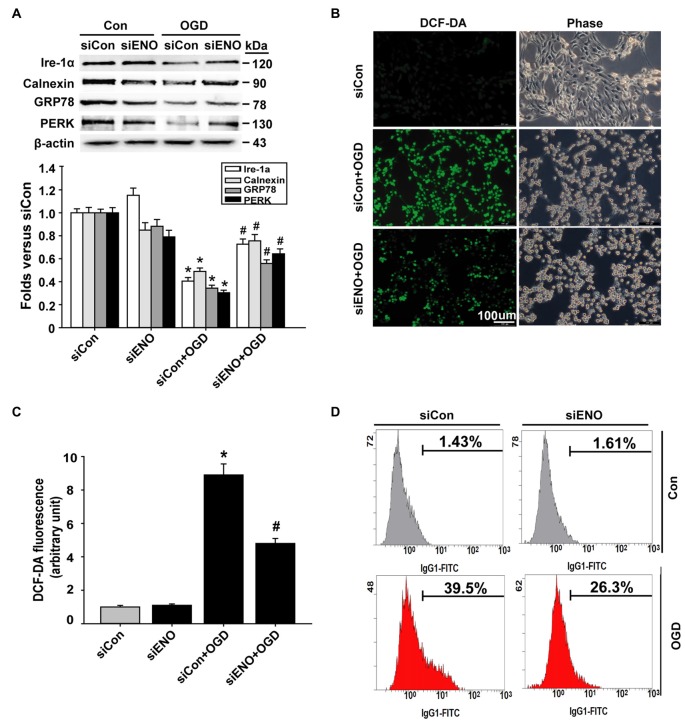
**Knockdown of ENOPH1 inhibits OGD-induced oxidative stress in bEND3 cells.** Down-regulation of ENOPH1 on OGD induced Endoplasmic reticulum (ER) stress signaling were determined by western blot. **(A)** Upper panel: representative immunoblots showing the changes of Ire-1a, calnexin, GRP78 and PERK protein bands in bEND3 cells after 6 h OGD treatment, with or without siENOPH1 transfection; bottom panel: quantitative data showed that OGD significantly decreased the mentioned ER stress chaperon protein level and knockdown of ENOPH1 inhibited this change. β-actin was used as a loading control. *P < 0.05 vs. siCon; #*P* < 0.05 vs. siCon + OGD; *n* = 4. **(B)** bEND3 cells transfected with siENOPH1 or control were loaded with DCF-DA (10 μM) to detect intracellular reactive oxygen species (ROS) production. Representative microscope pictures were shown (bar = 100 μm).** (C)** Quantification data showed that OGD significantly increased ROS production and knockdown of ENOPH1 inhibited ROS increase. *P < 0.05 vs. control; ^#^*P* < 0.05 vs. OGD; *n* = 4. **(D)** Representative flow cytometry of cells transfected with or without siENOPH1 after 6 h OGD treatment. Cells were stained with DCF-DA solution and then the levels of intracellular ROS were analyzed by flow cytometry; *n* = 4.

**Figure 6:**
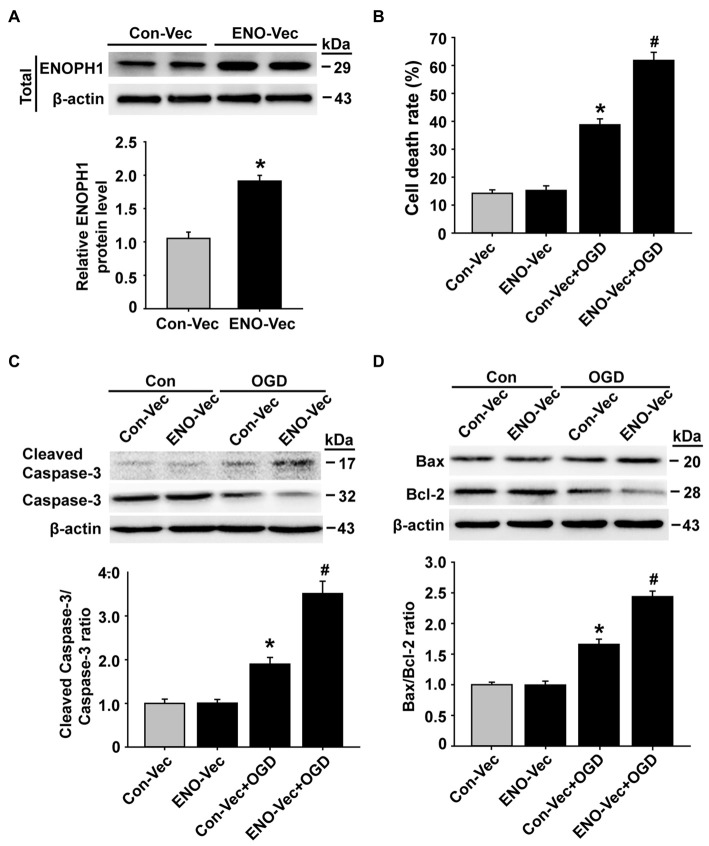
**Overexpression of ENOPH1 aggravates OGD-induced apoptosis in bEND3 cells. (A)** The ENOPH1 CRISPR activated plasmid effectively increased ENOPH1 protein expression in bEND3 cells. Upper panel: representative immunoblots showing the changes of ENOPH1 protein bands in bEND3 cells after 6 h OGD treatment, with or without ENOPH1 CRISPR activated plasmid (ENO-Vec) transfection; bottom panel: quantitative data showed that transfecting cells with ENO-Vec for 48 h significantly enhanced ENOPH1 protein levels *P < 0.05 vs. control activated plasmid (Con-Vec); *n* = 4. **(B)** ENOPH1 overexpression significantly increased 6 h OGD induced cell death assessed by lactate dehydrogenase release. *P < 0.05 vs. Con-Vec; ^#^*P* < 0.05 vs. Con-Vec + OGD; *n* = 4. **(C)** Upper panel: representative immunoblots showing the changes of cleaved and full length caspase-3 protein bands in bEND3 cells. β-actin was used as a loading control; bottom panel: quantitative data showed that OGD significantly increased the cleavage of caspase-3 and PARP and overexpression of ENOPH1 aggregated this change. *P < 0.05 vs. Con-Vec; ^#^*P* < 0.05 vs. Con-Vec + OGD; *n* = 4. **(D)** Upper panel: representative immunoblots of Bax and Bcl-2 proteins; bottom panel: quantitative data showed that OGD significantly increased the ratio of Bax/Bcl-2 and overexpression of ENOPH1 promoted this increase. *P < 0.05 vs. Con-Vec; ^#^*P* < 0.05 vs. Con-Vec + OGD; *n* = 4.

We next asked how ENOPH1 promoted microvascular endothelial cell injury after ischemic stimulation. Endoplasmic reticulum (ER) stress and oxidative stress are well-known mediators for BBB damage in ischemic stroke (Kaur and Ling, 2008; Yang et al., 2014). We speculated that ENOPH1 might contribute to ischemic bEND3 cell death/apoptosis through potentiating ER stress and oxidative stress under OGD conditions. To test this possibility, bEND3 cells were transfected with ENOPH1 siRNA before exposing the cells to OGD for 6 h. ER chaperone proteins (calnexin, PERK, Ire-1a and GRP78) were analyzed by western blot. As shown in Figure 5A, 6 h OGD induced a significant reduction in chaperone proteins calnexin, PERK, GRP78 and Ire-1a, and this reduction was significantly inhibited by knockdown of ENOPH1 with siRNA. ROS generation was assessed by DCFH staining and flow cytometry. As shown in Figures 5B,C, 6 h OGD induced a significant increase in DCFH fluorescence (green) intensity in bEND3 cells, indicative of increased ROS generation, which was inhibited by ENOPH1 siRNA, but not by control siRNA. Similar results were obtained when using flow cytometry to quantitate ROS generation in OGD-induced bEND3 cells transfected with ENOPH1 siRNA or control siRNA (Figure 5D). These results suggest that ENOPH1 may retard ER stress protective effect and potentiate ROS generation to promote endothelial cell death/apoptosis under OGD conditions.

### ENOPH1 Overexpression Enhances OGD-Induced Endothelial Injury and Oxidative Stress

To further verify an important role of ENOPH1 in potentiating endothelial cell death/apoptosis under OGD conditions, we transfected bEND3 cells with ENOPH1 CRISPR activation plasmid to elevate the ENOPH1 expression before exposing the cells to OGD treatment for 6 h and assessed its impact on cell death/apoptosis associated proteins. As shown in Figure 6A, western blot analysis showed that bEND3 cells transfected with ENOPH1 plasmid led to increase in ENOPH1 protein level at 48 h after transfection compared to control plasmid. Of note, ENOPH1 plasmid had no effect on cell death assessed by measuring 3-(4,5-Dimethylthiazol-2-yl)-2,5-diphenyltetrazolium bromide formation under control condition, but it significantly potentiated OGD-induced increase in cell death (Figure 6B). It also significantly enhanced OGD-induced activation of apoptosis-associated signal molecules, reflected by greater ratios of cleaved caspase-3/caspase-3 and Bax/Bcl-2 for OGD plus ENOPH1 plasmid-treated cells than OGD alone treated cells (Figures 6C,D). These data indicate that overexpression of ENOPH1 potentiates endothelial cell death/apoptosis under OGD conditions.

### ENOPH1 Regulates ADI1 Redistribution in Endothelial Cells Under OGD Condition

**Figure 7:**
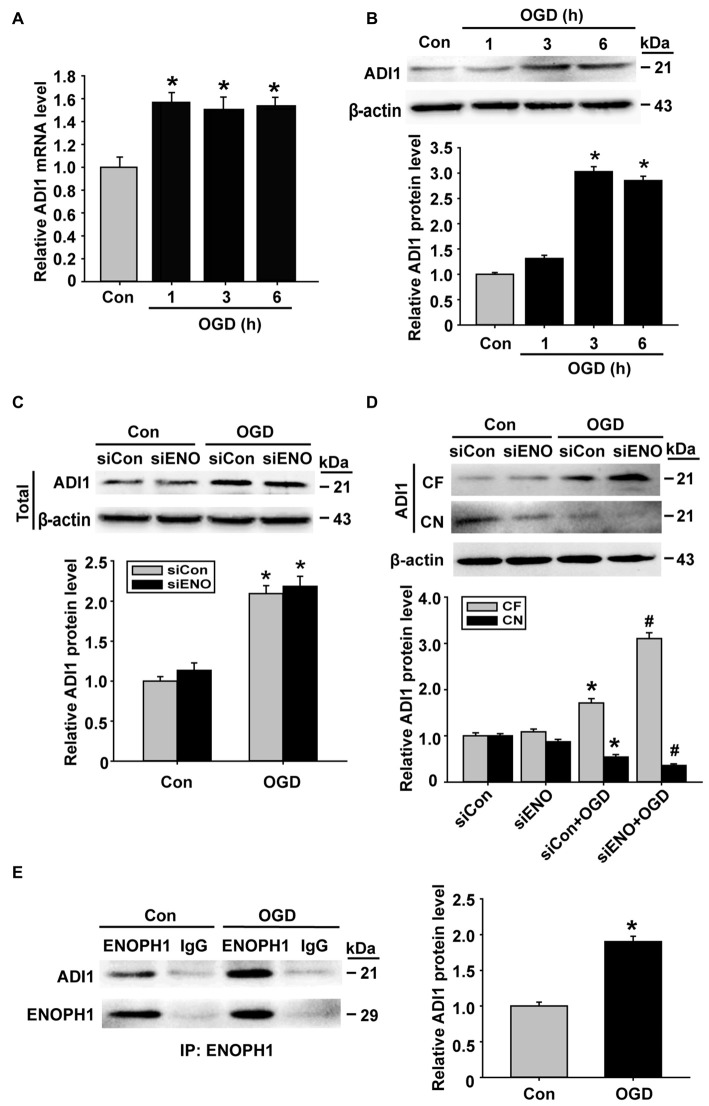
**ENOPH1 mediates OGD-induced ADI-1 relocation in endothelial cells. (A)** Real time RT-PCR analysis showed that aci-reductone dioxygenase 1 (ADI1) mRNA expression was significantly increased in bEND3 cells at 1 h after OGD treatment and was further increased when OGD was prolonged to 6 h. *P < 0.05 vs. Con; *n* = 4. **(B)** Western blot analysis showed that ADI1 protein levels were increased in 3 h OGD and 6 h OGD treated cells. Upper panel: representative immunoblots of ADI1 and the loading control β-actin; bottom panel: quantitative data of protein band intensity after normalization to β-actin., *P < 0.05 vs. Con; *n* = 4. **(C)** Upper panel: representative immunoblots of ADI1 and the loading control β-actin; bottom panel: quantitative data showed that transfected with ENOPH1 siRNA had not prevented OGD induced upregulation of total protein level of ADI in bEND3 cells. *P < 0.05 vs. siCon; *n* = 4. **(D)** Upper panel: representative immunoblots of ADI1 and the loading control β-actin; bottom panel: quantitative data showed that following OGD treatment, ADI1 levels in the CF was increased, while its level in CN was markedly reduced, which was enhanced by ENOPH1 siRNA. *P < 0.05 vs. siCon, ^#^*P* < 0.05 vs. siCon + OGD; CF, cytosolic fraction; CN, cytosolic nuclei; *n* = 4. **(E)** Left panel: representative immunoblots of coimmunoprecipitation of ENOPH1 and ADI1 from whole cell lysates of control cultures or OGD-treated cells with anti-ENOPH1 antibody or normal anti-IgG; right panel: quantitative data showed that OGD enhanced interaction of ENOPH1 with ADI1. *P < 0.05 vs. Con; *n* = 4.

ADI1 is a downstream protein of ENOPH1 and has been shown to play an important role in cell apoptosis, oxidoreductase reaction and virus infection (Hirano et al., 2005; Oram et al., 2007; Cheng et al., 2009). Therefore, we speculated that ADI1 might mediate the effect of ENOPH1 on OGD-induced endothelial cell death. To investigate this possibility, we studied the interaction between these two proteins. First, we examined the changes of ADI1 expression in OGD-treated bEND3 cells. As shown in Figure 7A, ADI1 mRNA expression was increased at 1 h after OGD treatment, which, unlike ENOPH1, was not further increased when OGD duration was prolonged to 3 h or 6 h. The change of ADI1 protein seemed to lag behind its mRNA, as bEND3 cells did not show increased ADI1 protein levels at 1 h OGD treatment and the increase in ADI1 protein was only seen after 3 or 6 h OGD (Figure 7B). To our surprise, knockdown of ENOPH1 with siRNA did not affect ADI1 protein expression under both normoxic and OGD conditions (Figure 7C), indicating that OGD-induced ADI1 upregulation was not ENOPH1-dependent. Interestingly, ENOPH1 knockdown significantly stimulated the translocation of ADI1 from the nuclei to the cytosol (Figure 7D). To demonstrate a direct interaction of ENOPH1 with ADI1, we conducted coimmunoprecipitation experiments. As shown in Figure 7E, ADI1 coimmunoprecipitated with ENOPH1 and OGD treatment enhanced the interaction of ENOPH1 with ADI1 by approximately one-fold over the control group (Figure 7E). Collectively, these data demonstrate that ENOPH1 might interact with ADI1 to restrict ADI1 in the nucleus and this interaction was enhanced under OGD conditions.

### Knockdown of ENOPH1 Reduces OGD-Induced Increase in the Permeability of bEND3 Cell Monolayer

**Figure 8:**
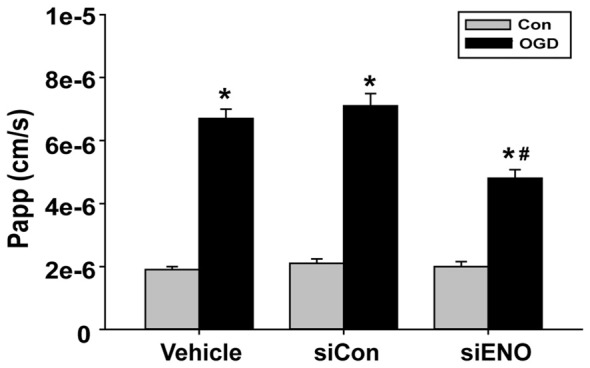
**Knockdown of ENOPH1 with siRNA reduces OGD induced blood brain barrier (BBB) disruption *in vitro*.** The permeability of fluorescein isothiocyanate (FITC)-dextran across bEND3 monolayers was significantly increased after 6 h OGD. This was partially inhibited by pretreating cells with ENOPH1 siRNA. The permeability of the endothelial monolayer was assessed by calculating the transfer rate of FITC-dextran from the luminal compartment to the abluminal compartment, and was expressed as the apparent permeability coefficient (Papp, cm/s). *P < 0.05 vs. Con, ^#^*P* < 0.05 vs. siCon + OGD; *n* = 4. Papp [cm/s] = dQ/(dt*A*Co), dQ: the amount of FITC-dextran getting into the abluminal compartment; dt : duration of OGD treatment; dQ/dt : the rate of transfer (ng/s); A: surface area (cm^2^); Co: the initial concentration in the luminal chamber (ng/cm^3^).

The above data have demonstrated a role of ENOPH1 in contributing to brain endothelial cell death under ischemic conditions, therefore we speculated that ENOPH1 was involved in OGD-induced endothelial barrier disruption. To test this possibility, we assessed the impact of ENOPH1 siRNA on the permeability of endothelial cell monolayer to FITC-dextran under control or OGD conditions. bEND3 cells were pre-treated with ENOPH1 siRNA before exposing to OGD for 6 h. As expected, 6 h OGD significantly increased the permeability of bEND3 cell monolayer to FITC-dextran (Figure 8). ENOPH1 siRNA, but not scrambled control siRNA, significantly reduced the permeability of OGD-treated endothelial monolayer to FITC-dextran (Figure 8). These results indicate that ENOPH1 is involved in OGD-induced loss of endothelial barrier function under ischemic conditions.

## Discussion

ENOPH1 is a newly identified enzyme of the methionine salvage pathway, and has been found to play a role in stress reactivity (Barth et al., 2014). In this study, we demonstrated a new role of ENOPH1 in mediating BBB injury under OGD conditions, which is a model of cerebral ischemia. The major findings include: (1) MCAO and OGD significantly increase ENOPH1 expression in cerebral microvessels and cultured brain endothelial cells (bEND3 cells), respectively; (2) knockdown of ENOPH1 with siRNA significantly attenuates OGD-induced bEND3 cell death and the activation of apoptosis-associated signal molecules, and accordingly overexpression of ENOPH1 with CRISPR-activated plasmid potentiates these changes; (3) knockdown of ENOPH1 attenuates OGD-induced ROS generation and ER stress; (4) OGD potentiates the interaction between ENOPH1 and its downstream molecule ADI1, increases ADI1 expression and promotes ADI1’s translocation into the cytosol; (5) ENOPH1 appears to restrain ADI1’s translocation from the nucleus to the cytosol, but does not affect OGD-induced ADI1 upregulation; and (6) knockdown of ENOPH1 attenuates OGD-induced endothelial barrier disruption.

Currently, the biological functions of ENOPH1 are largely unknown. A few recent studies have shown that ENOPH1 is widely expressed in the brain and is associated with neurodevelopmental disorders and anxiety (Barth et al., 2014; Komlósi et al., 2015). As the first study, here we investigated the role of ENOPH1 in ischemic stroke with a focus on the BBB. Our data show that both *in vivo* and *in vitro* ischemia induce ENOPH1 upregulation in cerebral microvascular endothelial cells. Of note, this change is quite rapid and persistent, as ENOPH1 mRNA expression was found to be increased in endothelial cells at 1 h after OGD treatment and remained high at the end of 6 h OGD exposure. Under our experimental conditions, significant cytotoxicity was only observed for 6 h OGD, but not for 1 h OGD and 3 h OGD, which was consistent with our previous studies (Liu et al., 2012, 2015). Therefore, in this study, 6 h OGD was chosen to test whether ENOPH1 contributes to brain endothelial cell death under ischemic conditions.

Our data show that knockdown of ENOPH1 with siRNA reduces OGD-induced cell death/apoptosis, while overexpressing ENOPH1 by transfecting ENOPH1 CRISPR activation plasmid results in a higher cell death rate in brain endothelial cells, which clearly supports a role of ENOPH1 in OGD-induced endothelial cell death/apoptosis. Moreover, along with ENOPH1 knockdown or overexpression is the inhibition or activation of apoptosis-associated proteins, including cleaved caspase-3, cleaved PARP, Bax and Bcl-2, further supporting a proapoptotic action of ENOPH1.

Increased free radical generation and impaired function of the (ER stress) are two common pathophysiological events occurring in the ischemic brain (Olmez and Ozyurt, 2012; Yu et al., 2016). Here, our data have also shown increased ROS generation in brain endothelial cells after 6 h OGD treatment. Of note, knockdown of ENOPH1 with siRNA markedly attenuated OGD-induced ROS generation in endothelial cells, suggesting that ENOPH1 may contribute to ischemic endothelial cell death via promoting intracellular ROS generation. Under ischemic conditions, there are several enzymatic and non-enzymatic sources of ROS generation, such as NADPH oxidase (Hur et al., 2010), the mitochondrial respiratory chain (Sanderson et al., 2013), and xanthine oxidase (Ono et al., 2009). In this study, we did not further explore the mechanism by which ENOPH1 enhances ROS generation under OGD condition. To our surprise, ischemic brain endothelial cells appear to have suppressed ER stress under our experimental conditions, as 6 h OGD led to suppressed expression of ER-stress associated proteins (Ire-1a, Calnexin, GRP78, and PERK). Moreover, ENOPH1 appears to act as an ER stress suppressor because knockdown of ENOPH1 significantly reverses OGD-induced suppression of ER stress-associated proteins. Our possible explanation for this unexpected observation would be ENOPH1 protein levels could retard ER stress protective effect by inhibiting some ER molecular chaperones (such as GRP78) expression in endothelial cells under OGD conditions.

ADI1 is a downstream molecule of ENOPH1 in methionine salvage pathway and has been shown to be implicated in cell apoptosis, cell growth inhibition, oxidoreductase reaction and virus infection (Hirano et al., 2005; Oram et al., 2007; Cheng et al., 2009). In addition, ADI1 is an oxidoreductase and can combine molecular oxygen donor to generate ROS (Oram et al., 2007). This known evidence has promoted us to hypothesize that ADI1 may mediate ENOPH1’s effect on promoting ischemic endothelial cell death. Our data show that OGD induces ADI1 mRNA and protein upregulation in a time-dependent manner similar to ENOPH1. To our surprise, although the coimmunoprecipitation assay clearly shows that ADI1 interacts with ENOPH1, knockdown of ENOPH1 does not affect OGD-induced ADI1 upregulation. Another interesting finding is that OGD increases the translocation of ADI1 from the nucleus to the cytosol and knockdown of ENOPH1 enhances this translocation. Our data raise several important questions: (i) what promotes the translocation of ADI1 to the cytosol under ischemic conditions; (ii) what translocated ADI1 does in the cytosol; and (iii) how ENOPH1 potentiates ADI1 translocation to the cytosol. Future studies are warranted to answer these questions.

Last, our data that knockdown of ENOPH1 partially inhibits OGD-induced permeability increase of the endothelial monolayer support an important role of ENOPH1 in ischemic BBB injury. It is well known that the brain capillary endothelial cells and the tight junctions between adjacent endothelial cells are the two most important structural components that maintain the integrity of the BBB (ElAli et al., 2011; Jumnongprakhon et al., 2016). Our current study was only focused the effect of ENOPH1 on ischemic endothelial injury and did not study its impact on the tight junctions. As a fact, ENOPH1’s downstream molecule ADI1 can bind to and inhibit the activity of membrane-type matrix metalloproteinase (MT1-MMP; Uekita et al., 2004; Chang et al., 2016), and our data demonstrate that ENOPH1 enhances the translocation of ADI1 to the nucleus under OGD conditions. As thus, there is a great possibility that ENOPH1 may act as a separator to push ADI1 away from the MT1-MMP (located in cell membrane and the cytosol), and thus activate MT1-MMP. The latter in turn activates MMP-2 to mediate the degradation of tight junction proteins and the components of the basal membrane, leading to BBB disruption (Yang et al., 2007). Future studies are needed to test this possibility.

Taken together, our present study defines a novel role of ENOPH1 in ischemic brain injury, in which cerebral ischemia triggers ENOPH1 upregulation in brain endothelial cells, leading to increased ROS generation and the activation of apoptosis associated molecules to contribute to ischemic cell death. Moreover, under OGD condition, ENOPH1 promotes the translocation of its downstream molecule ADI1 from the cytoplasm to the nucleus, future studies are needed to investigate the mechanisms underlying this change and its biological consequence.

## Author Contributions

Conceived and designed the experiments: WL (Wenlan) and YZ. Performed the experiments: YZ, TW and KY. Analyzed the data: YZ, JX and LR. Contributed reagents/materials/analysis tools: TW and KY. Wrote the article: WL (Wenlan) and WL (Weiping).

## Funding

The work was supported by grants from National Natural Science Foundation of China (81571149); by China Postdoctoral Science Foundation (2015M572387), and Project Funded by Shenzhen Science and Technology Innovation Commission (JCYJ20150330102401097, KQCX20140521101427034, ZDSYS20140509173142601).

## Conflict of Interest Statement

The authors declare that the research was conducted in the absence of any commercial or financial relationships that could be construed as a potential conflict of interest.

## Abbreviations

BSA: Bovine albumin
DAPI: 4′,6-diamidino-2-phenylindole
DCF-DA: 2,7-dichlorodihydrofluorescein diacetate
EDTA: Eathylene diamine tetraacetic acid
FACS: Fluorescence-activated cell sorting
FITC: fluorescein isothiocyanate
PBS: Phosphate Buffered Saline
TUNEL: terminal deoxynucleotidyl transferase-mediated dUTP-biotin nick end labeling.
